# Efficient Measurement of Opsonising Antibodies to *Plasmodium falciparum* Merozoites

**DOI:** 10.1371/journal.pone.0051692

**Published:** 2012-12-26

**Authors:** Danika L. Hill, Emily M. Eriksson, Amandine B. Carmagnac, Danny W. Wilson, Alan F. Cowman, Diana S. Hansen, Louis Schofield

**Affiliations:** 1 Walter and Eliza Hall Institute for Medical Research, Parkville, Victoria, Australia; 2 Department of Medical Biology, University of Melbourne, Parkville, Victoria, Australia; Universidade Federal de Minas Gerais, Brazil

## Abstract

**Background:**

Antibodies targeting merozoites are important in protection from malaria. Therefore, merozoite surface proteins are attractive vaccine candidates. There is a need for robust functional assays to investigate mechanisms of acquired immunity and vaccine efficacy. To date, the study of merozoite phagocytosis has been confounded by the complexity and variability of *in vitro* assays.

**Methodology/Principal findings:**

We have developed a new flow cytometry-based merozoite phagocytosis assay. An optimized merozoite preparation technique produced high yields of merozoites separated from haemozoin. Phagocytosis by the undifferentiated THP-1 monocytic cell line was mediated only by Fc Receptors, and was therefore ideal for studying opsonising antibody responses. The assay showed robust phagocytosis with highly diluted immune sera and strong inter-assay correlation. The assay effectively measured differences in opsonisation-dependent phagocytosis among individuals.

**Conclusions/Significance:**

This highly reproducible assay has potential applications in assessing the role of opsonic phagocytosis in naturally acquired immunity and vaccine trials.

## Introduction

Naturally acquired immunity to *Plasmodium falciparum* malaria develops over time and exposure, involving both humoral and cell mediated immune responses. Immunity is non-sterilizing and results in reduced parasite densities and protection from clinical disease [1]. Antibodies, particularly IgG1 and IgG3 subclasses, are crucial components of acquired immunity and develop against surface antigens of sporozoite, intra-erythrocytic and merozoite forms of the asexual life cycle[1]–[3]. The importance of antibodies for protection against clinical episodes of *P. falciparum* malaria was highlighted by passive transfer experiments where γ-globulin from immune African adults afforded protection against severe malaria to non-immune children [4], [5].

Identifying antibody responses to the merozoite surface coat in human studies has typically involved ELISA-based serology. However conflicting findings have been reported for various antigens, with some studies reporting associations between antibody levels and protection from disease, while others do not [6]. ELISA methodologies do not discriminate the large proportion of immunoglobulin produced during infection that may bind antigen or peptide *in vitro* but may be functionally irrelevant. Furthermore, antibody affinity and avidity, and the role of antibody-leukocyte cooperation, are not measured using ELISA endpoints. Such serology alone provides only limited information about antigenic targets of acquired immunity. Thus there is a need for assays better able to measure functionally protective responses and their antigenic targets.

Currently the only functional assays that have been applied to the study of acquired immunity to *P. falciparum* merozoites are *in vitro* growth inhibition assays [7], [8]. Growth inhibition assays, which in part are considered to measure merozoite invasion inhibition, have not always revealed associations with clinical immunity [9]–[11]. They also do not examine interactions between antibody and cellular immunity. Numerous antigens produce an opsonising antibody response that requires leukocyte co-operation for anti-parasitic functions [12]–[14]. Furthermore, several vaccines under development, such as MSP3-LSP, may require antibody-leukocyte co-operation to be efficacious [15].

An Antibody Dependent Cellular Inhibition (ADCI) assay has been used for measurement of opsonising antibody responses [16]. This assay has led to identification of clinically important antigens such as merozoite surface protein 3 (MSP-3) [14] and GLURP [13]. In passive transfer experiments, protective immune plasma inhibited parasite growth *in vitro* only in the presence of monocytes in the ADCI assay [16]. However, various limitations have hampered widespread application of this assay to clinical and research settings, and associations between assay outcomes and clinical protection are not yet proven. The basis of the assay depends on IgG:monocyte interaction where cytophilic IgG is essential, resulting in the release of a soluble factor from monocytes which inhibits the growth of surrounding intra-erythrocytic parasites [17]. Antibody function is then measured by reduction in parasite viability, as assessed by giemsa stained blood smears [16], [18], and more recently by flow cytometry [19], [20]. In combination with primary monocytes and the use of purified IgG, the resulting assay is time consuming, variable and quite complex. These factors may contribute to the lack of reproducibility reported for this assay in different settings [21].

Like ADCI, phagocytosis of merozoites also requires cytophillic IgG and Fc Receptors (FcR). The importance of phagocytosis in malaria was demonstrated by macrophage depletion in mice, which abolished acquired immunity despite unchanged antibody profiles [22]. In human studies, phagocytic opsonising antibody responses to mature parasitized red blood cells are associated with reduced risk of placental malaria in primigravidae, secundigravidae and HIV-infected individuals [23], [24]. Monocytes, macrophages and neutrophils also phagocytose merozoites both *in vitro* and *in vivo* [25]–[29]. Merozoites are highly exposed to the immune system and a strong antibody response is mounted against the merozoite surface coat. Human monoclonal antibodies to a merozoite surface antigen with strong invasion inhibition activity *in vitro*, failed to transfer immunity to mice unless recipients were transgenic for appropriate FcR [30]. Thus, phagocytosis of merozoites before they invade red blood cells (RBC) may also be a mechanism for controlling parasitemia in immune humans.

However, merozoite phagocytosis has been poorly studied, and it remains unclear whether antibodies promoting phagocytosis of merozoites confer protection and reduce parasite burden in humans. A major difficulty lies in the reproducibility of *in vitro* merozoite phagocytosis assays. This appears to results from i) difficulty in isolating intact and viable merozoites for use in *in vitro* assays, ii) donor variability in primary phagocytic cells [31], and iii) difficulty in discerning FcR- from non-FcR-mediated phagocytosis. Collectively, these factors make current merozoite phagocytosis assays difficult to standardize and apply to cohort studies and clinical trials for the analysis of association with parasitological and clinical risk.

To address these limitations we have developed a simplified phagocytosis assay to investigate the functional activity of human antibodies. A recently developed isolation technique, adapted to phagocytosis assays, enabled purification of large yields of fluorescent merozoites separated from haemozoin. Utilising undifferentiated THP-1 cells allowed highly reproducible measurement of phagocytosis of opsonised merozoites mediated by Fc Receptors [32], [33]. These improvements, in addition to a flow cytometry readout, have resulted in a method returning high inter-assay reproducibility and sufficient throughput that is suitable for vaccine trials and for large longitudinal cohort studies.

## Materials and Methods

### Collection of Plasma and Serum Samples

Plasma samples utilized for this study were collected from children (5–12 yrs) and adults (18–53 yrs) from Madang Province on the north coast of Papua New Guinea (PNG). The study was approved by the Medical Research Advisory Committee, Papua New Guinea Ministry of Health, The Walter and Eliza Hall Institute Human Research Ethics Committee (project number 04/04). Written consent was obtained from parents/guardians of all participants. Non-immune plasma from anonymous healthy Western Australian adult donors was isolated from whole blood donated by the Australian Red Cross Blood Service.

### THP-1 Monocyte Cell Line

The human monocytic cell line THP-1 (TIB-202, American Type Culture Collection (ATCC) was maintained in RPMI-1640 supplemented with 10% foetal bovine serum (FBS) and 55 µM 2-mercapthoethanol (THP-1 medium), and maintained below a density of 5×10^5^ cells/mL.

### *P. falciparum* 3D7 Culture and Merozoite Isolation

*P. falciparum* 3D7 parasites were cultured at 4% hematocrit in RPMI-1640 supplemented with 25 mg/mL HEPES, 2 mg/mL sodium bicarbonate, and 10% pooled human serum (parasite medium). Cultures were maintained at 37°C in an atmosphere of 5% CO_2_, 1% O_2_ and 94% N_2_, and synchronized using 5% sorbitol. Late-stage parasites (36–40 hr) were isolated (>95% purity) from uninfected RBCs with a MAC magnet separation column (Miltenyi Biotech). Schizonts were treated with Epoxysuccinyl-L-leucylamido(4-guanidino)butane (E64), as described previously [34]. Briefly, parasites were incubated with 10 µM E64 (Sigma-Aldrich) overnight for up to 12 hrs. Schizonts were pelleted at 1,900×g for 8 min, and resuspended in 3 mL THP-1 media and filtered through a 1.2-µm Acrodisc 32-mm syringe filter (Pall). Filtered merozoites were separated from haemozoin by passing the 3 mL suspension twice over an equilibrated LS MACS Column (Miltenyi Biotech), followed by a final wash of the column with 1.5 mL THP-1 media. Merozoites were stained with ethidium bromide (EtBr) for 30 mins (10 µg/mL, Bio-Rad) at room temperature, followed by two washes in 10 mL THP-1 medium, with centrifugation at 4,000×g for 8 mins. Merozoites were resuspended in 3 mL THP-1 medium, and absolute number determined using Count-Bright Absolute Counting Beads (Invitrogen) as per manufacturer’s protocol.

### Phagocytosis Assay

EtBr-stained merozoites were suspended in 150 µL THP-1 medium per well and incubated in FBS-coated 96-well U-bottom plates with serum or plasma samples for 40 mins at 22°C in the dark. Various merozoite:THP-1 cell ratios were tested ranging from 50∶1 to 1∶1. Different plasma dilutions ranging from 1/10 to 1/5×10^−8^ were also used. Following opsonisation, 50 µL aliquots of merozoites were resuspended and transferred to FBS-coated 96-well U-bottom plates. THP-1 cells were added at a final concentration of 5×10^5^/mL to make up a final volume of 200 µL per well and each sample was tested in triplicate. Plates were incubated for 40 mins at 37°C in 5% CO_2_ 1% O_2_ and 94% N_2_ humidified incubator. Phagocytosis was arrested by centrifugation at 500×g for 5 min at 4°C. Cells were washed twice in ice-cold PBS and fixed in 100 µL 2% paraformaldehyde (PFA). Samples were acquired (20,000 events) using a FACSCalibur (BD Bioscience) flow cytometer with HTS plate reader attachment. Viable cells were gated by forward and side scatter. In experiments where inhibition of phagocytosis was performed, THP-1 cells were treated with cytochalasin D at 10 µM for 90 mins prior to addition to free merozoites. In FcR blocking experiments, THP-1 cells were incubated with 10 ug/ml of non immune human IgG (Sigma), for 45 mins prior to addition of free merozoites. Percentage phagocytosis refers to the % of EtBr positive cells with immune plasma minus the % positive cells with non-immune plasma.

### FITC Stained Merozoite Phagocytosis Assay and Quenching of Surface Fluorescence

Following filtration and removal of haemozoin, merozoites were centrifuged at 4000×g and stained with 0.1 mg/mL FITC in PBS for 30 mins at 37°C. Merozoites were washed twice in 15 mL THP-1 medium, and resuspended in 3 mL THP-1 medium. The concentration of merozoites was determined using Count-Bright Absolute Counting Beads (Invitrogen) as described above. FITC merozoites were opsonised and phagocytosed as per EtBr phagocytosis assay above, fixed in 50 µL 2% PFA and kept at 4°C until acquisition. To quench surface FITC fluorescence, an equal volume of trypan blue buffer (0.1 M citrate buffer pH4.0 with 250 µg/mL trypan blue) was added and cells incubated on ice for 1 minute prior to acquisition. THP-1 cells stained with CD14-FITC (clone M5E2, BD Biosciences) were used as a positive control for quenching efficiency. Samples were acquired (20,000 events) using a FACSCalibur (BD Bioscience) flow cytometer.

### Anti-sera, SDS-PAGE and Immunoblot Analysis

Anti-MSP-3 and anti-MSP-6 anti-sera were raised in mice against full length purified proteins. Rabbit anti-sera to MSP-1_19_ was generated as described previously [35]. Rabbit AMA-1 anti-sera was a gift from A. Hodder. 3D7 schizonts, merozoites after filtration and after EtBr staining were separated on 4–12% SDS–NuPAGE gels (Invitrogen) and transferred to PVDF membrane (Invitrogen). Reactvity of anti-MSP-3, MSP-6 and AMA-1 was compared by immunoblotting and detection by anti-rabbit or mouse IgG horseradish peroxidase (HRP) conjugate (Millipore), and visualised via enhanced chemiluminescence (ECL, Amersham Biosciences).

### Indirect Immunofluorescence Assay (IFA)

3D7 merozoites were washed twice and fixed with 0.0075% glutaraldehyde/4% PFA (ProSciTech, Australia) for 30 mins at 22°C and air-dried onto slides. Fixed parasites were subsequently blocked overnight in 3% BSA (Sigma) in PBS at 4°C. Parasites were then incubated with rabbit antisera to MSP-3 and MSP-1_19_ in 3% BSA/PBS for 1 hr at 22°C. Following two washes in PBS, parasites were stained with Alexa Fluor-594 conjugated anti-mouse secondary antibody (Invitrogen) for 1 hr at 22°C. After three washes in PBS, parasites were mounted in VectaShield® (Vector Laboratories) with 0.1 ng/µL 4′,6-diamidino-2-phenylindole, DAPI (Invitrogen). Fluorescence images were obtained using a Plan-Apochromat 100×/1.40 oil immersion Phase contrast lens (Zeiss) on an AxioVert 200 M microscope (Zeiss) equipped with an AxioCam Mrm camera (Zeiss). Images were processed using Photoshop CS5 (Adobe).

### Statistics

Data were analysed using Graph Pad Prism v5. For comparisons between different THP-1 cell treatment conditions, a two-tailed student t-test was performed with 95% confidence. For comparison of inter-assay variability, a non-parametric Spearman correlation and Bland-Altman mean-difference test were performed with 95% confidence intervals.

## Results

### Merozoite Isolation Technique Removes Haemozoin and Maintains Merozoite Surface Integrity

**Figure 1:**
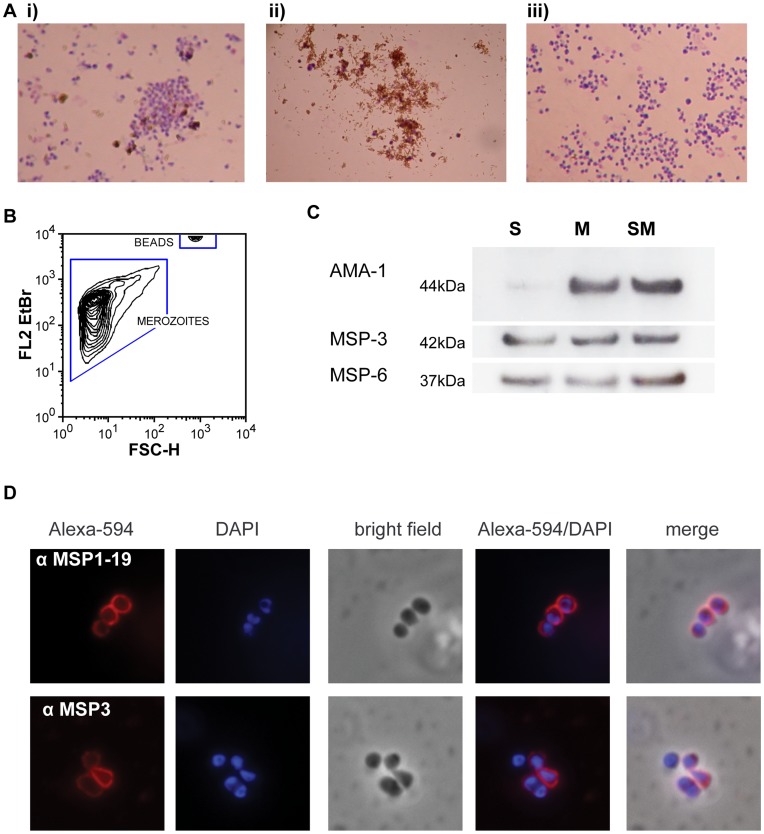
Isolated merozoites maintain surface coat integrity. A) E64-treated schizonts were filtered to release free merozoites and haemozoin crystals, and the filtrate was passed over magnetic columns. Merozoite purification was confirmed by Giemsa-stained smears of i) filtrate, ii) retained haemozoin and iii) purified merozoites. B) Merozites were stained with EtBr and enumerated by flow cytometry. C) Washed Merozoites retained surface proteins MSP-3, MSP-6 and AMA-1 by western blot (S: purified Schizonts; M: filtered merozoites; SM: EtBr stained merozoites). D) Merozoite surface proteins are maintained during merozoite isolation and wash steps as shown by surface localisation of MSP-3 and MSP1_19_ by immunofluorescence microscopy. Antigens were stained with Alexa-594 and the nucleus with DAPI (panels in order Alexa549; DAPI; brightfield; Alexa549/DAPI; merge).

When added to late trophozoite stages, the protease inhibitor E64 prevents schizont rupture, resulting in parasitophorous vacuolar membrane enclosed merozoite structures, or PEMS [36]. A recent method utilizes E64 to generate PEMS, which are ruptured by filtration to release viable merozoites and haemozoin crystals [37]. We have adapted this method to produce large yields of merozoites for subsequent use in phagocytosis assays. The presence of haemozoin crystals in solution with merozoites promoted formation of large aggregates of haemozoin and merozoites. Haemozoin can be removed by passing the filtrate over magnetized columns [34], and we have refined this technique to enable isolation of merozoites free of haemozoin for phagocytosis assays (Figure 1a). To quantify uptake of merozoites into THP-1 cells, merozoites were stained with EtBr and enumerated by flow cytometry using fluorescent counting beads (Figure 1b).

We sought to determine whether the integrity of the merozoite surface coat was impaired by multiple washing steps required for EtBr staining. Western blot and immunofluorescence assay confirmed peripherally associated merozoite surface proteins MSP-3 and MSP-6 were not shed or lost during washing steps (Figure 1c and 1d). In addition, GPI-anchored MSP-1_19_ and transmembrane AMA-1 were also maintained. Detailed investigations of rhoptry, microneme and merozoite surface protein expression in filtered merozoites have been reported elsewhere [34], [37].

### Ethidium Bromide Fluorescence Marks Internalised Merozoites within THP-1 Cells

**Figure 2:**
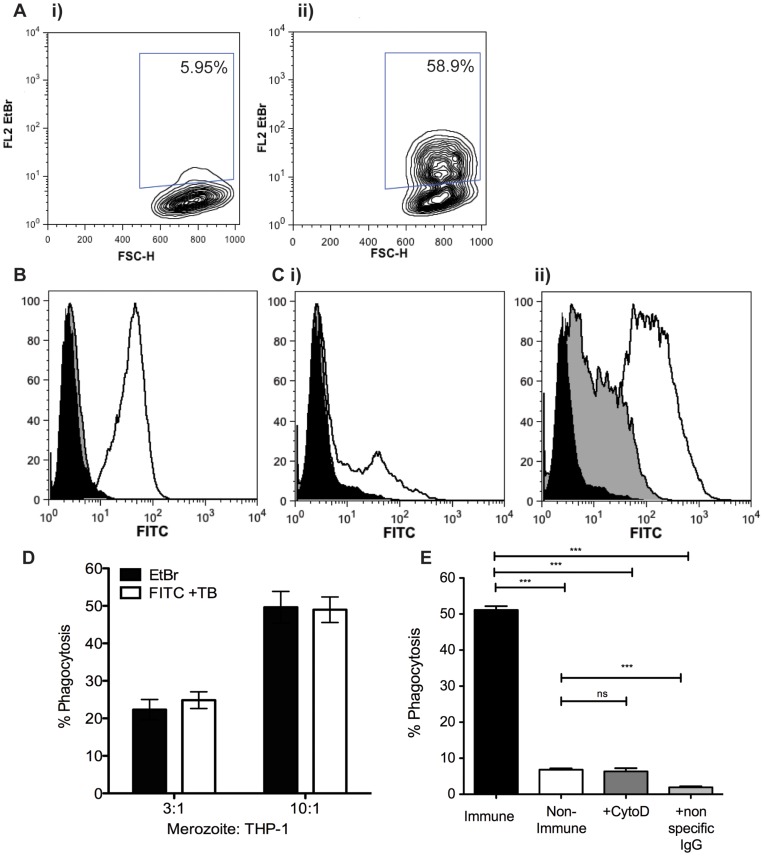
Phagocytosis by THP-1 cells is antibody and Fc Receptor dependent. A) EtBr stained merozoites were incubated with i) non-immune plasma or ii) immune PNG plasma, and were added to THP-1 cells. THP-1 cells were gated by forward and side scatter, and EtBr fluorescence was determined by flow cytometry. A non-immune control sample was used to set the EtBr positive gate. B) THP-1 cells were stained with anti-CD14 antibody and treated with trypan blue (TB) buffer which quenched all surface FITC fluorescence (black:THP-1 cells only, white: CD14-FITC stained THP-1, grey: CD14-FITC stained THP-1 with TB buffer). C) FITC stained merozoites were opsonised with i) non-immune or ii) immune PNG plasma and added to THP-1 cells. FITC fluorescence was measured by flow cytometry before and after quenching, and phagocytosed merozoites were resistant to quenching (black:THP-1 cells only, white: fluorescence with FITC stained merozoites, grey: fluorescence with FITC stained merozoites after TB buffer). D) The % phagocytosis measured for EtBr stained merozoites was equivalent to FITC stained merozoites after quenching. Opsonised EtBr stained and FITC stained merozoites were added to THP-1 cells at 3∶1 and 10∶1 merozoite:THP-1 ratios. E) Phagocytosis is active and Fc Receptor dependent. THP-1 cells were treated with cytochalasin D (CytoD) or blocked with non-specific IgG prior to addition to immune plasma opsonised merozoites in the phagocytosis assay. Each point represents the mean ± standard error. *, *p*<0.05; **, *p*<0.01; ***, *p*<0.005.

A strong phagocytosis response was observed for merozoites opsonised with immune plasma, with low levels of background EtBr positive cells observed with non-immune plasma (Figure 2a). Fluorescence with non-immune plasma was consistently equivalent to non-opsonised controls, and hence merozoites opsonised with non-immune plasma were included as a negative control in each experiment. A phagocytosis response was also observed using GFP expressing D10 parasites [38], demonstrating application to other lines of *P. falciparum* (data not shown), however utilizing an EtBr stain allows the assay to be adapted to any parasite line or isolate. To test whether merozoites were phagocytosed by THP-1 cells and not adherent to the cell surface, isolated merozoites were FITC-stained and phagocytosis assay performed, and an acidic trypan blue (TB) buffer added prior to flow cytometry [39]. TB has been shown to quench only surface FITC staining, providing a useful tool to differentiate adherent from internalized particles [40]. To confirm that TB buffer was able to quench surface FITC fluorescence from THP-1 cells, monocytes were stained with FITC-conjugated anti-CD14 antibody. Incubation of monocytes with TB buffer efficiently quenched FITC fluorescence associated with surface staining using this antibody (Figure 2b). To confirm antibody-dependent phagocytosis, merozoites were then stained with EtBr or FITC and utilized in the phagocytosis assay (Figure 2a and c). When FITC stained merozoites incubated with non-immune plasma were added to THP-1 cells and quenched by TB buffer, fluorescence was indistinguishable from THP-1 cells alone (Figure 2c). Following quenching, the FITC mean fluorescence intensity (MFI) also decreased in THP-1 cells added to immune plasma opsonised merozoites. However, the percentage of phagocytosis observed with FITC-quenched THP-1 cells added to immune plasma opsonised merozoites, after non-immune background subtraction, was comparable to the percentage of phagocytosis demonstrated with EtBr stained merozoites at the ratio of 3∶1 and 10∶1 merozoites:THP-1 cells (Figure 2d).

### Phagocytosis of Merozoites is an Active Process Requiring Fc Receptor Mediated Interactions

Cytochalasin D is an actin polymerization inhibitor, which prevents cytoskeletal rearrangements necessary for phagocytosis. THP-1 cells were treated with Cytochalasin D and EtBr fluorescence was reduced to a level equivalent to the non-immune serum controls (Figure 2e), indicating active phagocytosis. To determine the importance of Fc Receptor phagocytosis, FcR were blocked with high concentrations of non-specific IgG. FcR blocked THP-1 cells showed a complete reduction in phagocytic capacity, indicating FcR-antibody interaction is the underlying mechanism for merozoite phagocytosis observed (Figure 2e).

### Phagocytosis Assay Optimisation

**Figure 3:**
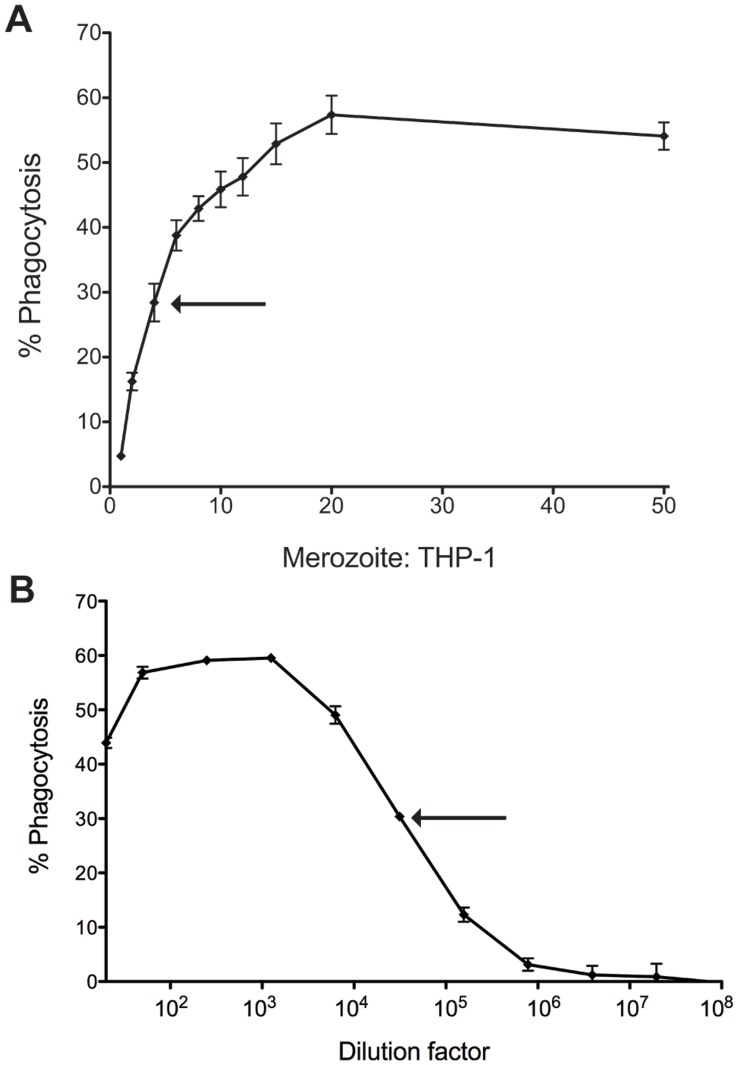
Efficient merozoite phagocytosis at low antibody concentrations. A) Increasing numbers of EtBr labeled merozoites were incubated with a pool of immune plasma from PNG children and then added to THP-1 cells. The % phagocytosis was determined by flow cytometry. The ratio of 4∶1 was chosen for subsequent assays, and is indicated by the arrow above. B) Titration of a pool of immune plasma from PNG children for opsonising activity using the 4∶1 merozoite:THP-1 cell ratio. % phagocytosis was determined by flow cytometry. The chosen dilution, 1/30,000, is indicated by an arrow above.

THP-1 cells are very efficient phagocytes for latex particles, platelets, erythrocytes and trophozoites [33], [41]–[43]. Different particle-to-THP-1 cell ratios have been described in phagocytosis assays. The number of merozoites required was optimized, and ratios from 1∶1 to 50∶1 merozoite:THP-1 cells were tested. Merozoite phagocytosis was very robust in the presence of immune plasma, with phagocytosis measurable as low as one merozoite per THP-1 cell. Phagocytosis reached a maximum at 20∶1 merozoites:THP-1 cell. The lower dilution of 4∶1 merozoites:THP1 was chosen for subsequent assays as this is within the dynamic range useful for detecting individual differences between diverse sample sets (Figure 3a).

### Assay Sensitivity and Reproducibility

**Figure 4:**
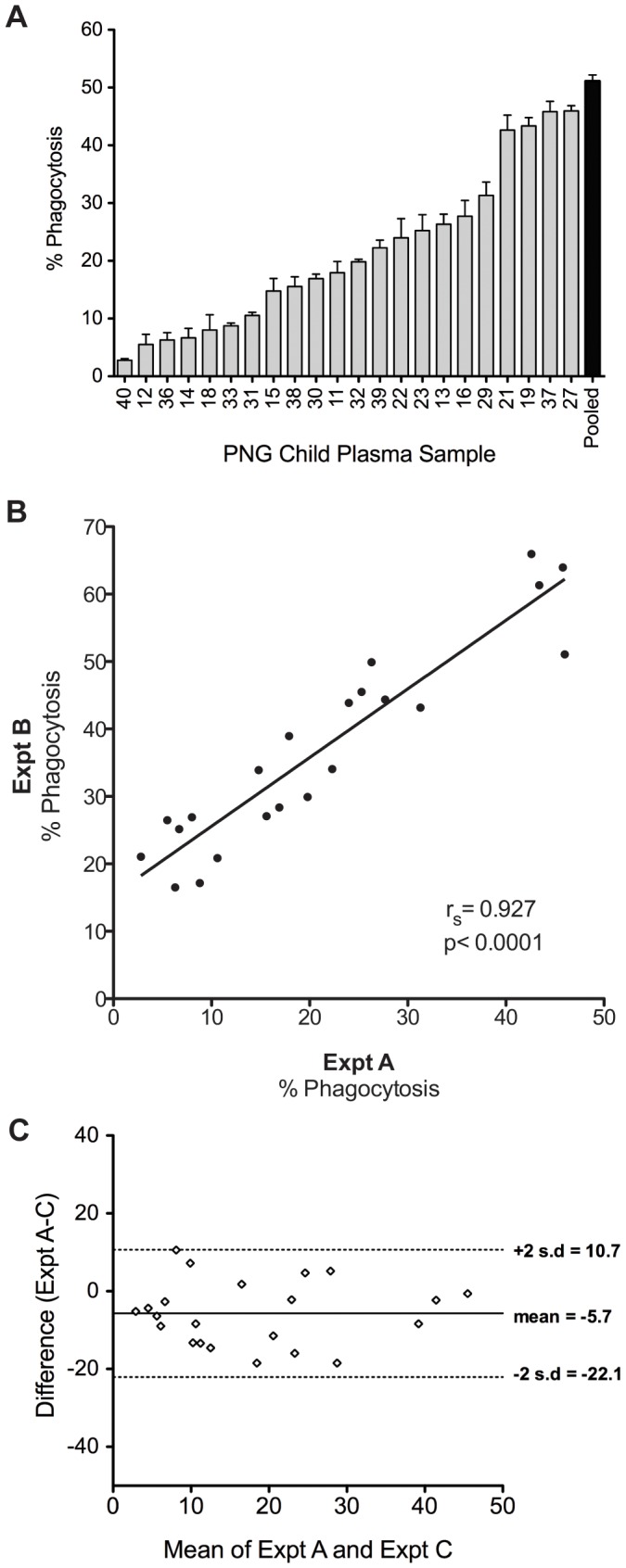
Optimised Phagocytosis Assay is sensitive and reproducible. A) Percentage phagocytosis for 22 plasma samples from PNG children, compared to a pooled PNG adult positive control. B) Interassay comparisons as determined by Spearman Correlation, or (C) Bland-Altman test.

Plasma volumes were titrated to determine the dynamic range in which variation in responses between individuals could be detected. Serial dilutions of a pool of plasma from 22 PNG children were performed and the phagocytosis readout determined (Figure 3b). High concentrations of plasma caused a reduction in phagocytosis capacity consistent with the pro-zone effect [44], an effect known to influence THP-1 phagocytosis [33]. The dynamic range of the assay occurred between 1/6000 and 1/150,000 dilution. The optimal dilution chosen was 1/30,000 as phagocytosis in this range was still robust but was positioned half way between the maximum and minimum responses. Using these optimized assay conditions, plasma samples from 22 PNG children aged 5–10 years old were tested. A 1/50 dilution of PNG adult plasma was used as a positive control for maximal responses. These assay conditions enabled resolution of differences between individuals, detecting low, high or intermediate responses (Figure 4a). When these plasma samples were utilized on different experimental days with different batches of THP-1 cells and merozoites, we detected outstandingly reproducible and correlated responses by Spearman’s correlation (Figure 4b, r_S_ = 0.927, p<0.0001) and Bland-Altman test (Figure 4c).

### Assay Detects Rapid Opsonisation

**Figure 5:**
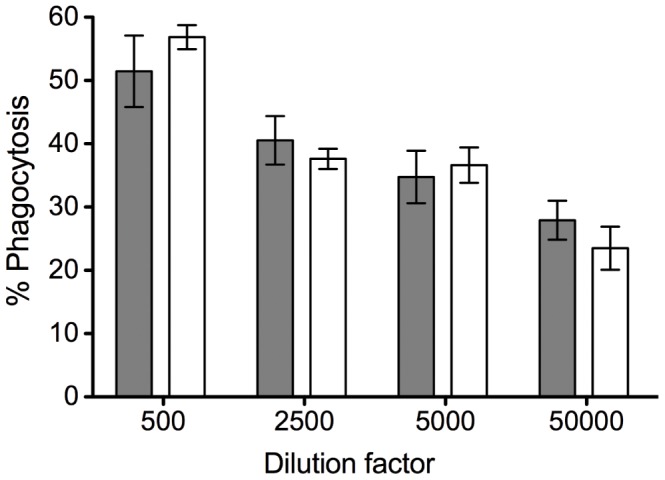
Merozoite opsonisation proceeds rapidly at low antibody concentrations. Merozoites were added to wells containing THP-1 cells simultaneously (white bars), or after 40 mins of preincubation (grey bars), with varying dilutions of an immune plasma pool. % phagocytosis was determined by flow cytometry.

To further assess the efficacy of opsonisation, the pool of PNG child plasma was tested at several dilutions with or without a pre-incubation step (i.e. merozoites and plasma were pre-incubated or added simultaneously to wells containing THP-1 cells). At the four dilutions tested, no difference was observed between the two conditions (Figure 5). These findings further highlight that merozoite opsonisation leading to phagocytosis occurs very rapidly and is highly efficient.

## Discussion

Merozoite surface proteins are attractive malaria vaccine candidates due to their exposure to the immune system, and the ability of antibodies directed to such surface antigens to inhibit parasite development. Humoral responses against merozoite surface antigens can function to inhibit invasion directly [45], [46] or to opsonise merozoites leading to phagocytosis [29], respiratory burst [47] or ADCI [48]. Functional immunological assays that measure these key effector mechanisms are more likely to reveal associations with clinical immunity than serology alone [49]. Several vaccines are in development that may require leukocyte co-operation for vaccine-induced antibodies to provide clinical protection [50], [51]. Thus, there is a clear need for better assays to test the potential protective efficacy of these mechanisms.

Phagocytosis is a complementary mechanism to ADCI for cytophillic IgG to produce an antibody mediated cellular protective response against merozoites. There has been limited study in this area, however, due to the difficulties in isolating merozoites from culture supernatants. Merozoites isolated in this way are collected over several hours of schizont rupture and extensive handling and washing steps are required. The technique optimized here enables a high yield of fluorescently labeled merozoites, with surface coat intact, to be isolated by filtration and magnetic separation from haemozoin. Haemozoin has long been studied for its ability to modulate immune responses both with stimulatory [52]–[55] and inhibitory [56], [57] effects. These effects are especially pronounced in monocytes which rapidly phagocytose haemozoin, leading to rapid oxidative burst [52] whilst inhibiting repeat phagocytosis [58]. In addition to these effects, haemozoin promotes the formation of merozoite-haemozoin aggregates, and hence it is critical to perform haemozoin free merozoite phagocytosis assays.

Previous investigations of merozoite phagocytosis have used mostly microscopy readouts [25]–[28], or FITC stained merozoites and flow cytometry [29]. EtBr staining is more specific for merozoites than protein stains such as FITC, allowing confidence that fluorescence is due to intact merozoites and not other parasite and red blood cell material.

Previous merozoite phagocytosis assays and current ADCI assays use primary monocytes or neutrophils. Primary human phagocytic cells display variable capacities for phagocytosis, exhibit FcR polymorphisms, and can be modulated by exposure to inflammatory cytokines *in vivo.* These factors limit the utility of primary phagocytes in *in vitro* assays to measure antibody functions. THP-1 cells have been used extensively in phagocytosis assays after chemical differentiation [59], [60], however these cells perform non-FcR mediated phagocytosis [61]. Undifferentiated THP-1 cells are non-adherent enabling flow cytometric readout [41], [59], [60]. Undifferentiated THP-1 cells only phagocytose through Fc receptors, where FcγRI, FcγRIII, and all isoforms of FcγRII are expressed [32], [33], [62]. As all phagocytic Fc receptors are expressed, THP-1 present an ideal cell type for investigating opsonising antibodies and the contribution of Fc receptors in merozoite phagocytosis. As demonstrated in this paper, the THP-1 cell line can robustly and reproducibly phagocytose merozoites, but only in the presence of immune plasma.

Using the optimized phagocytosis assay, merozoite phagocytosis was very efficient and required minimal amounts of antibody. Saturated phagocytosis was observed using more than a hundred times less plasma than existing studies of merozoite phagocytosis [27], [29], or growth inhibitory assays (GIA) [63], [64]. Significant further dilution was required to reach a non-saturating range, using a 1/30,000 dilution. This requirement for dilute concentrations of plasma to elicit a functional response from monocytes is within the range observed using ADCI, where as low as 700 pM IgG inhibited parasite growth [17]. This finding stresses the importance of determining the dynamic range for *in vitro* antibody functional assays that serve as a correlate for immunity. IgG2, IgG4 and IgM have been reported to inhibit binding of cytophillic IgG to malarial antigens [65]–[67]. As this assay utilizes plasma, rather than purified IgG, it enables measurement of opsonizing responses in the presence of inhibiting antibody isotypes and subclasses, which are present *in vivo*.

As noted, functional immunity to *P. berghei* requires phagocytes [22], and appropriate FcRs are required for passive transfer of immunity by human monoclonal antibodies directed towards the merozoite antigen MSP-1 [30]. In humans, *P. falciparum* mature parasites sequester, hence merozoite egress and invasion occurs in deep vascular beds (not in peripheral circulation) where they may come into contact with effector cells. As merozoite invasion of red cells can occur rapidly, phagocytosis must also be highly efficient if it is to be protective. We have shown that opsonisation leading to merozoite phagocytosis is rapid and efficient, but the contribution of merozoite phagocytosis to immune protection remains to be determined. Although the degree of merozoite phagocytosis was found to increase with immune status in a few African individuals [25], the causal link between this functional response and decreased disease risk has not been rigorously tested. The merozoite phagocytosis assay described here has sufficient throughput and minimal requirements for plasma to allow use in large cohort studies in order to determine relative risk and to assess vaccine efficacy and immunogenicity.
